# Yolk Sac Tumor of the Omentum: A Case Report and Literature Review

**DOI:** 10.3390/diagnostics12020304

**Published:** 2022-01-25

**Authors:** Daniela Fischerova, Tereza Indrielle-Kelly, Andrea Burgetova, Rosalie Jana Bennett, Maria Gregova, Pavel Dundr, Ondrej Nanka, Giulia Gambino, Filip Frühauf, Roman Kocian, Martina Borcinova, David Cibula

**Affiliations:** 1 Department of Obstetrics and Gynecology, First Faculty of Medicine, Charles University and General University Hospital in Prague, 128 51 Prague, Czech Republic; Filip.Fruehauf@vfn.cz (F.F.); roman.kocian@vfn.cz (R.K.); martina.borcinova@vfn.cz (M.B.); david.cibula@vfn.cz (D.C.); 2 Department of Obstetrics and Gynecology, Burton Hospitals NHS, West Midlands DE13 0RB, UK; t.indriellekelly@gmail.com; 3 Department of Radiology, First Faculty of Medicine, Charles University and General University Hospital in Prague, 128 08 Prague, Czech Republic; Andrea.Burgetova@vfn.cz; 4 Department of Pathology, First Faculty of Medicine, Charles University and General University Hospital in Prague, 128 08 Prague, Czech Republic; Rosalie.Bennett@vfn.cz (R.J.B.); maria.gregova@vfn.cz (M.G.); pavel.dundr@vfn.cz (P.D.); 5 Institute of Anatomy, First Faculty of Medicine, Charles University, 128 00 Prague, Czech Republic; ondrej.nanka@lf1.cuni.cz; 6 Department of Gynecology and Obstetrics, University of Parma, 43126 Parma, Italy; gambinogiulia@hotmail.it

**Keywords:** endodermal sinus tumor, yolk sac, ovary, omentum, diagnostic imaging, biopsy, ultrasonography, female

## Abstract

This is a case report of a rare finding of an extragonadal yolk sac tumor in a 37-year-old patient who presented with shortness of breath and abdominal bloating. During imaging and staging surgery, the findings were strongly suggestive of an extragonadal advanced tumor presenting with peritoneal dissemination, predominantly affecting omentum, with no clear primary origin. Histology revealed an extragonadal yolk sac tumor in a pure form outside the ovaries. Lacking an obvious origin elsewhere, the tumor was highly suspected to have truly originated from the omentum. The patient underwent surgery and four cycles of chemotherapy consisting of cisplatin, etoposide, and bleomycin. One-year outpatient follow-up thereafter showed no relapse. We herein discuss a possible site of the tumor origin and its development, as well as diagnostic challenges and disease prognosis.

## 1. Introduction

Yolk sac tumors (also known as primitive endodermal tumors or endodermal sinus tumors) are malignant germ cell neoplasms that typically arise from the ovary or testis. In females, yolk sac tumors typically present in childhood or early adulthood; the median age of diagnosis is 19 years, with 40% of patients diagnosed in the prepubertal period [1]. They are highly malignant, rapidly growing tumors with a very brief duration of symptoms and which metastasize rapidly on the peritoneum, on the lymph nodes, and via the hematogenous route [2]. Ascites can be present on imaging [2]. 

Approximately one-third of yolk sac tumors are of extragonadal origin, with origins such as the vulva, vagina, cervix, endometrium, sacrococcygeal area, pelvis, retroperitoneum, mediastinum, and others [3,4]. Extragonadal yolk sac tumors represent a diagnostic challenge, particularly due to various sites of origin influencing the signs and symptoms of the disease [5,6].

In this work, we report a case of a 37-year-old female patient with an extragonadal yolk sac tumor, FIGO stage IIIC [7], which presented with nodular peritoneal involvement extending from the pelvis to the upper abdomen, including omental cake, and without deep infiltration of the viscera or a clear site of origin. 

## 2. Case Presentation

**Figure 1:**
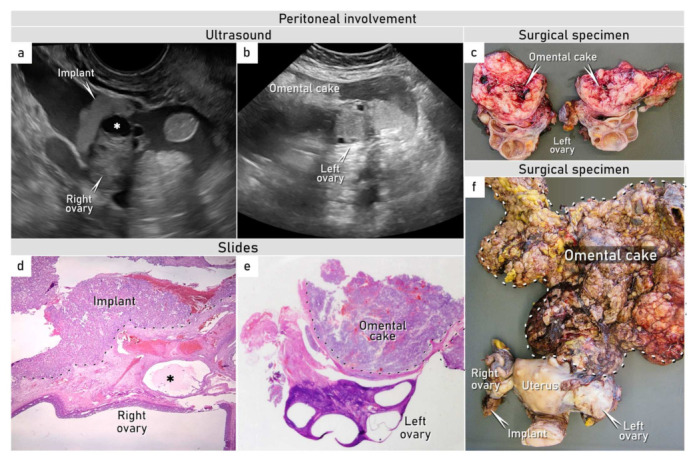
Peritoneal involvement. Ultrasound images in transverse plane demonstrating (**a**) peritoneal visceral implant on the right ovary and (**b**) omental infracolic cake extending on the visceral surface of the left ovary and uterine fundus. (**c**) Surgical specimen showing infracolic omentum densely adherent to the left ovary. (**d**) Microscopic specimen (H&E, 40×) showing tumor implant secondarily infiltrating the cortex of the right ovary; the dashed line shows the boundary between the superficial tumor implant and the ovarian cortex. (**e**) Omental cake is adherent to the surface of the left ovary on histotopogram without any neoplastic involvement of the ovary itself; the dashed line shows the boundary of the infracolic omental cake in adhesions to the left ovarian surface. (**f**) Surgical specimen of uterus and ovaries. See also Supplementary Video S1. * Follicular cyst found on the ultrasound examination (**a**) and in microscopic specimen (**d**).

A 37-year-old patient was referred for an expert ultrasound at the gynecologic oncology center for a suspicious pelvic mass. She presented with a 2-week history of abdominal bloating, nausea and vomiting, and shortness of breath on exertion. On physical examination, her abdomen was distended with marked ascites, and there was a large abdomino-pelvic mass palpable on bimanual vaginal examination. Apart from two Caesarian sections in the past, she was fit and well with a normal BMI. Her father died of prostate cancer aged 75, and her brother underwent treatment for testicular cancer at the age of 39. An ultrasound revealed intact ovaries covered by peritoneal implants on the right side and by omental cake on the left side (Figure 1). The implants were mainly present as isolated nodules which showed slight hyperechogenicity with smooth outlines.

**Figure 2:**
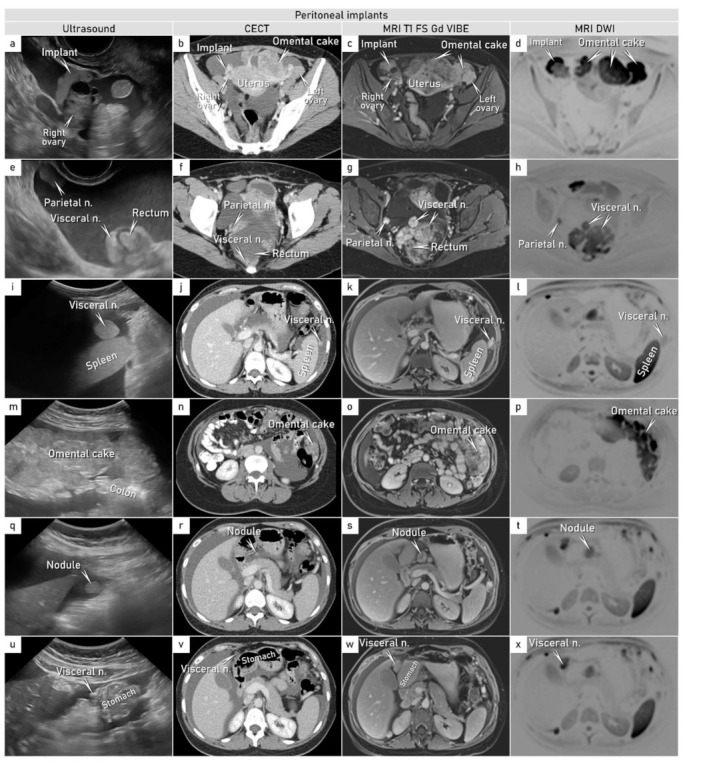
Peritoneal involvement (pelvis and abdomen). Ultrasound, contrast-enhanced computed tomography, and magnetic resonance with diffusion-weighted images demonstrating peritoneal metastases in transverse plane (unless stated otherwise): (**a**–**d**) Peritoneal visceral implant on the right non-infiltrated ovary. (**e**–**h**) Parietal isolated nodule on the right pelvic side wall and diffuse visceral peritoneal involvement on rectosigmoid. (**i**–**l**) Visceral focal nodule on the splenic surface. (**m**–**p**) Diffuse omental infiltration (omental cake). (**q**–**x**) Visceral nodules in the omental bursa and on the stomach (in sagittal plane on ultrasound). Abbreviations: n., nodule(s); MRI T1 FS Gd VIBE, T1-weighted magnetic resonance imaging after intravenous gadolinium; VIBE, volumetric interpolated breath-hold examination; CECT, contrast-enhanced computed tomography; DWI, diffusion weighted imaging. Supplementary Video S1.

Implants were also found on the rectosigmoid serosa, but without any deep infiltration of the viscera. Furthermore, there was an extensive omental cake in the supracolic region reaching the spleen and in the infracolic region reaching the pelvis, with an isolated visceral nodule on the splenic and stomach surfaces and within the omental bursa, all associated with ascites and fluidothorax (Figure 2).

To address possible non-ovarian origins of the tumor, an ultrasound-guided transvaginal tru-cut biopsy was performed at the end of the ultrasound examination, and a blood sample was taken for CEA (carcinoembryogenic antigen), CA 19-9 (carbohydrate antigen 19-9), CA 125 (cancer antigen 125), and CA 15-3 (cancer antigen 15-3) to narrow down possible primary origins. Tumor markers were normal and the tru-cut biopsy showed papillary and tubular arrangements of a tumor consisting of cells with marked nuclear atypia, with frequent mitoses and clear or eosinophilic cytoplasm (Supplementary Figure S1).

Immunohistochemically, the tumor cells showed expression of SALL4 (sal-like protein 4), HNF1B (hepatocyte nuclear factor 1-beta), PAX8 (paired box gene 8), and α-FP (alpha fetoprotein). Cytokeratin 7 was positive in rare cells. EMA (epithelial membrane antigen) was negative (Supplementary Figure S2).

The tru-cut biopsy showed unusual morphology (clear cell carcinoma-like pattern) and an overlapping immunoprofile between a yolk sac tumor and clear cell carcinoma. The pathognomic Schiller–Duval bodies frequently present in yolk sac tumors were absent in the tru-cut biopsy [8]. Based on a possible gynecological origin of tumor spread and following ISAAC (Imaging Study in Advanced Ovarian Cancer) study protocol (ClinicalTrials.gov, NCT03808792) designed for the description of suspected primary ovarian, tubal, or peritoneal cancer, additional cross-sectional imaging was added, i.e., chest and abdomen contrast-enhanced computed tomography (CECT) and whole-body diffusion-weighted magnetic resonance imaging (WB-DWI/MRI) (Figure 2). Cross-sectional imaging confirmed the ultrasound findings. 

The results of the imaging, tumor markers, and tru-cut biopsy were discussed in a multidisciplinary tumor board meeting. Tumor resectability was demonstrated on review of the preoperative imaging findings, and due to the worsening of a patient’s condition requiring frequent drainage of ascites, a decision was made for upfront debulking surgery without repeated biopsy or additional diagnostic tests. Intraoperative findings showed a frail omental tumor with contact bleeding fixed to the anterior abdominal wall and fused with uterine fundus and left adnexa (Figure 1). 

**Figure 3:**
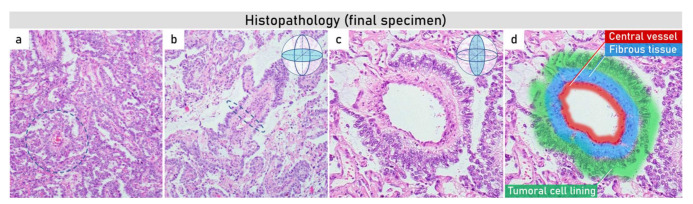
Histopathology (final specimen). The specimens (**a**,**b**) at 200× *g* magnification demonstrate (**a**) papillary pattern combined with small tubopapillary endodermal sinus structure (Schiller–Duval body) in blue circle; (**b**) marked tubulopapillary sinusoidal structure with central vascular core in longitudinal section (Schiller–Duval body); (**c**,**d**) 400× *g* magnified image plus zoom of diagnostic round cystic Schiller–Duval body in a transverse section, with microcystic and papillary patterns around. The body has a central vessel surrounded by fibrous tissue, called the fibrovascular core, and it is surrounded by layers of the tumoral cells at the surface of that stalk. The structure is located in open cystic space also lined by tumoral cells. All those structures together are called a Schiller–Duval body and resemble primitive glomerulus.

A total abdominal hysterectomy with a bilateral salpingo-oophorectomy was performed with a pelvic peritonectomy, total omentectomy, and partial diaphragmatic stripping and extirpation of abdominal peritoneum with no macroscopic residual disease. Histological evaluation of the final specimen showed typical morphological features of a yolk sac tumor, including Schiller–Duval bodies (Figure 3).

The tumor was classified as a possibly extragonadal yolk sac tumor with a neoplastic involvement of the omentum, pelvic, and abdominal peritoneum, including ovarian serosa on the left side; incipient infiltration of the ovarian cortex on the right side; and positive cytology findings, categorizing it as FIGO stage IIIC (Figure 1) [7]. After surgical treatment, serum α-FP was analyzed, and its level reached 495.6 ng/mL (normal range for adults: 10–20 ng/mL). In order to exclude very rare gastric yolk sac tumors, a gastroscopy was performed, which was normal. Other primary tumor origins, such as mediastinum or retroperitoneum, were not detected on the whole-body imaging and/or exploration. The origin of the tumor remained unclear, with the majority of the tumor mass in the omentum. 

The patient underwent adjuvant chemotherapy consisting of four cycles of bleomycin, etoposide, and cisplatin (BEP regimen), with serum α-FP levels normalizing by the third cycle (Figure S3). Following this, serum α-FP remained within the normal range 12 months after the initial diagnosis (the latest follow-up visit at the outpatient unit). The patient will continue regular follow-up at the outpatient unit; the serum α-FP levels will be monitored every 3 months for the first 2 years after treatment, every 6 months during the third year, and then in 12-month intervals [9]. No severe short-term or long-term toxicity of chemotherapy was observed.

## 3. Discussion

Yolk sac tumors are the second most common type of malignant germ cell tumors that can occur in both genders [10]. Regarding the female tract, they can be divided into several categories. The first group is represented by congenital tumors, often admixed with teratomas. The second group are tumors in prepubertal children that are more commonly pure. In postpubertal children and adults, yolk sac tumors are usually mixed with other germ cell tumors. Finally, in older adults, so-called “somatically derived yolk sac tumors” usually arise in ovaries, also in association with another tumor, such as high-grade serous carcinoma or clear cell carcinoma [11]. This subset of yolk sac tumors in older adults most likely arises via retrodifferentiation, by which the differentiated cells transform into a more primitive form [11]. There is growing evidence that these somatically derived yolk sac tumors, which are usually diagnosed at more advanced stages, may have worse prognosis and that their response to standard chemotherapy is not as clear as in true germline yolk sac tumors in young patients [12]. One of the distinguishing features between somatic and germline origin is zygosity. Complete homozygosity suggests postmeiotic germ cell origin even in adult patients [13].

Ovarian yolk sac tumors are often detected at an early stage in young women, usually in the second or third decade of life, presenting with pain due to very rapid growth and markedly elevated serum α-FP. On ultrasound, malignant ovarian yolk sac tumors are mostly unilateral, large, and multilocular-solid or solid, with fine-textured slightly hyperechogenic solid tissue and rich vascularization [14]. On MRI, yolk sac tumors can show areas of signal voids related to rich vascularization [15] or areas of hemorrhage. Cross-sectional imaging is valuable in the assessment of the full extent of metastatic spread into the lymph nodes, omentum, lungs, liver, and bones [16]. In advanced stages, surgical treatment aims to provide complete cytoreduction without systematic lymph node dissection; however, the procedure may be modified to minimize surgical morbidity in view of high chemosensitivity to ensure no delays in postoperative chemotherapy [9]. Given the very high chemosensitivity of yolk sac tumors, potential nodal metastasis should be targeted by adjuvant chemotherapy in these patients, and nodal dissection should be carried out only in the case of nodal abnormality [9]. Platinum-based regimens are the treatment of choice, with the BEP regimen being the most widely used; generally, four cycles of a 5-day BEP regimen are considered to be the standard. However, in a completely resected disease of stage I, II, or III with no macroscopic residual disease and negative tumor markers after surgery, three cycles of BEP may be administrated with adequate safety and efficacy [17,18]. 

The tumor marker associated with 60–90% of yolk sac tumors is alpha fetoprotein (α-FP), and its normalization with chemotherapy is regarded as an indication of complete clinical response to the treatment [10,19,20]. Follow-up visits must include history, tumor markers’ analysis (α-FP; lactate dehydrogenase; CA125), and physical examination including pelvic examination. The visits should be performed in 3-month intervals for the first 2 years post-treatment, in 6-month intervals during the third year, and then yearly until progression. 

The prognosis of gonadal yolk sac tumors is usually very good, especially owing to the early stage of the disease, chemoresponsive nature of the tumors, and low age of the patients. Several classification systems have been developed to individualize the prognosis assessment and to triage patients with germ cell tumors for appropriate chemotherapy regimens [19]. Most recently, the International Germ Cell Cancer Collaborative Group has introduced an outlook-factor-based consensus classification, which includes the primary site, concentrations of pretreatment tumor markers, and extent of the disease [19]. There are three risk groups according to this classification system (good, intermediate, and poor). The highest risk of recurrence and chemotherapy failure is associated with a primary site in the mediastinum, visceral non-pulmonary metastases, and high serum tumor markers (especially α-FP ˃ 10,000 ng/mL).

Extragonadal germ cell tumors are rare, with an incidence of 1.8–3.4 in 1,000,000 in the female population [20]; they can be found in the majority of structures along the midline, starting from the brain (up to 46%) to the coccyx [1]. In the literature, two main theories of extragonadal yolk sac tumors have been proposed. According to the first theory, the tumor originates from the aberrant differentiation of somatic cells, which may explain yolk sac tumors occurring in the stomach, endometrium, or lung [21]. The second theory suggests that the tumor originates from primitive germ cells which are either truly of extragonadal origin or which represent metastatic spread from the gonads. Traditionally, the hypothesis of etiopathogenesis of extragonadal germ cell tumors relied on the malignant transformation of primordial germ cells disseminated erroneously at the midline during migration in the first gestational trimester [1]. However, an alternative theory by McKenney et al. argued that extragonadal tumors may represent a spread from occult undiagnosed or regressed malignant lesions in the gonads [22]. This is supported by occasional findings of “regressive” features in peripheral foci with signs of scarring [23]. 

This case report described a patient with a yolk sac tumor presenting with a nodular peritoneal involvement extending from the pelvis to abdomen, diffusely consuming omentum—but without any clear deep infiltration of underlying organs, including the ovaries, and without a clear primary origin of the tumor. By revision of the histopathology, neither regressive features supporting a hypothesis of regressed malignant lesion in gonads nor somatically derived yolk sac tumors (absence of high-grade serous or endometrioid carcinoma) were detected. Similarly, in addition to whole-body imaging, postoperatively performed gastroscopy also excluded aberrant differentiation of somatic cells elsewhere (stomach, lung etc.), further supporting the presence of a true extragonadal germline yolk sac tumor. 

Primordial germ cells first develop outside the embryo in the wall of the yolk sac, close to the allantois. As the embryo develops, they migrate along the thin tissue of the dorsal mesentery of the hindgut to the posterior abdominal wall, where in the genital ridge, they become part of the gonads [24]. In embryos of 6 weeks or later, almost all primordial germ cells already accumulate in the gonads [24]. Deviation from precise migratory program of these embryonic tissues may explain the extragonadal origin of germ cell tumors along the line of migration towards the genital ridges [24]. 

**Figure 4:**
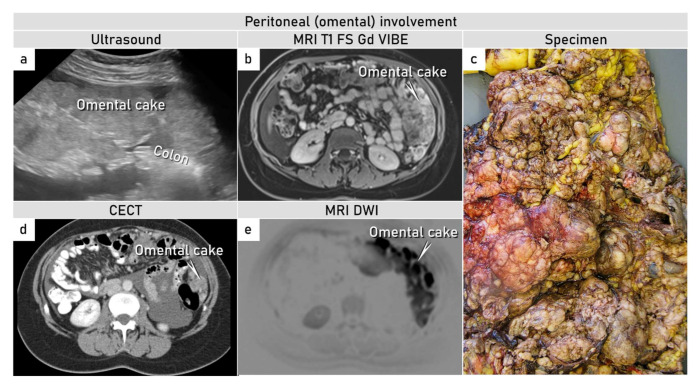
Omental tumor. (**a**–**e**) Ultrasound, magnetic resonance with diffusion-weighted images, contrast-enhanced computed tomography, and specimen demonstrating omental tumor (images in transverse plane). (**a**–**e**) Omental cake. Abbreviations: MRI T1 FS Gd VIBE, T1-weighted magnetic resonance imaging after intravenous gadolinium; VIBE, volumetric interpolated breath-hold examination; CECT, contrast-enhanced computed tomography; DWI, diffusion-weighted imaging.

Therefore, we believe that the tumor of the described patient originated from the malignant transformation of primordial germ cells misplaced during their migration from the yolk sac through dorsal mesentery involving greater omentum, which embryologically developed from the rotation of dorsal mesogastrium. With the lack of obvious origin elsewhere, a yolk sac tumor predominantly affecting omentum and spreading onto surrounding structures is highly suspected to truly originate from the omentum (Figure 4). 

In total, there are eight cases of primary omental yolk sac tumors reported in the literature to date, summarized in Table 1 [25,26,27,28,29,30,31,32]. Generally, the omentum is an extremely rare location of this tumor. All the published cases show similarities to our case, with a rapid onset of symptoms (mainly abdominal distension) and findings of an abdominal mass possibly originating from the omentum on CT, MRI, or ultrasound. 

The inclusion of α-FP among tumor markers prior to surgery reflects the consideration of this rare pathology as a differential diagnosis. It was pivotal in the diagnosis of six omental tumors out of the eight published cases (nine cases, including our case report; 67%). Not including the α-FP level, the diagnosis would rely on a histopathologic diagnosis from an intraoperative frozen section or a small amount of tissue from a tru-cut biopsy, which could be inconclusive since the presence of Schiller–Duval bodies is only found in 20% of yolk sac tumors [28]. The main differential diagnoses of yolk sac tumors include clear cell carcinoma and embryonal carcinoma and can be complicated, especially in limited samples. In doubtful cases, immunohistochemistry can be very helpful. 

In all reported cases, the maximum effort was made to achieve a complete cytoreduction, and in all but one, at least four cycles of a cisplatin-based chemotherapy combination, including cisplatin, etoposide, and bleomycin, were applied in either an adjuvant or a neoadjuvant setting. It is remarkable that this rare extragonadal primary omental yolk sac tumor was mostly reported in Asia (7/9 patients, 78%), which can be associated with the higher prevalence of germ cell tumors in Asia [33]. 

Symptoms of the presented case, such as increased abdominal size, persistent abdominal bloating, abdominal or pelvic (lower belly) pain, feeling full after eating a small amount, needing to urinate often or urgently, were mimicking ovarian cancer [5,6], with prompt referral to a gynecologic oncology center. On ultrasound, the appearance of malignant peritoneal involvement differed from epithelial malignant ovarian cancers. Peritoneal involvement by the yolk sac tumor demonstrated homogeneous echogenicity and presented as isolated nodules of slightly increased homogenous echogenicity and smooth outlines (Supplementary Video S1). In comparison, high-grade serous carcinomas usually show non-homogeneous echogenicity, predominantly hypoechogenic, with nodules merging together forming a plaque-like appearance of varying (non-uniform) thickness and irregular outlines. There were no intensely hyperechogenic spots indicative of psammoma bodies, which are typically seen in implants related to low-grade serous cancer [34]. In addition, mucinous cancers, both primary and metastatic from the primary gastrointestinal tract site, usually present as hypo- to isoechogenic solid nodules in combination with loculated fluid [35]. Other malignant epithelial cancers (endometrioid, clear cell carcinoma, carcinosarcoma, etc.) usually have similar appearances to high-grade serous cancers. The atypical appearance of peritoneal involvement and intact ovaries led the sonographer to perform the tru-cut biopsy in order to facilitate the diagnosis [36,37,38]. However, the limited amount of fragmented tissue together with unusual morphology and overlapping immunoprofile made the pathologists unable to differentiate the yolk sac tumor from clear cell carcinoma. 

Instead of repeated biopsy, the upfront debulking surgery was initiated and was successful in achieving no residual disease, but α-FP remained high postoperatively, which is why the patient was scheduled for four cycles of BEP (not three cycles) in line with NCCN (National Comprehensive Cancer Network) guidelines [18]. The elimination of α-FP within the third cycle of chemotherapy can be considered as a prognostically positive factor. For the assessment of the prognosis of extragonadal yolk sac tumors, the International Germ Cell Cancer Collaborative Group classified the prognosis of our patient as intermediate, with estimated 5-year progression-free survival at 75% and 5-year overall survival at 80% after surgical staging and adjuvant chemotherapy. The prognosis was considered due to the fact that our patient did not have visceral (intraparenchymal) and/or lymph node metastasis or postoperative residual tumor, and her postoperative α-FP was 495.6 ng/mL. At the latest follow-up, one year post surgery, the patient remained in complete remission.

## 4. Conclusions

This case report demonstrates a rare occurrence of a primary omental yolk sac tumor with peritoneal spread and ascites. Ultrasound in combination with raised level of serum α-FP can help discriminate peritoneal involvement by the yolk sac tumor from carcinomatosis. The histological diagnosis is established on the typical morphological and immunohistochemical profile. 

## Figures and Tables

**Table 1:** Review of primary yolk sac tumors of the omentum. Abbreviations: CT, chemotherapy; BEP, bleomycin, etoposide, and cisplatin; CR, complete clinical response. * Neoadjuvant CT (chemotherapy) composed of intravenous cyclophosphamide and arterial cisplatin and doxorubicin.

Author	Symptoms	Age at Diagnosis (Gender)	Occurrence	Level of α-FP (ng/mL)	Preoperative Biopsy	Surgery	Chemotherapy	Follow-Up	Year of Publication
Park et al. [25]	Abdominal distension	45 (female)	Asia (Korea)	20,250 (preoperatively)	No	Total abdominal hysterectomy, bilateral salpingo-oophorectomy, infracolic omentectomy	Adjuvant BEP (4×)	CR (10 months)	1999
Xinghui et al. [26]	Abdominal distension	3 (male)	Asia (China)	>1210 (preoperatively)	Yes	Omentectomy	Neoadjuvant CT *	Not available	2004
Geminiani et al. [27]	Abdominal pain	46 (female)	Europe (Italy)	21,550 (preoperatively)	No	Hysterectomy, bilateral salpingo-oophorectomy, omentectomy, resection of bowel with terminal ileostomy.	Adjuvant BEP (4×)	CR (24 months)	2005
Kim et al. [28]	Lower abdominal pain and distension	37 (female)	Asia (Korea)	2980 (postoperatively)	No	Supracolic omentectomy, total abdominal hysterectomy, bilateral salpingo-oophorectomy, multiple peritoneal biopsies, cytology, pelvic and paraaortic lymph node dissection, appendectomy	Adjuvant BEP (4×)	CR (12 months)	2009
Haibin et al. [29]	Abdominal distension	44 (female)	Asia (China)	27,612 (preoperatively)	No	Abdominal hysterectomy with bilateral salpingo-oophorectomy and infracolic omentectomy	Adjuvant BEP (4×)	CR (7 months)	2010
Harano et al. [30]	Abdominal distension.	35 (male)	Asia (Japan)	7144 (preoperatively)	Yes	Neoadjuvant CT (BEP) for 4 cycles + surgery (omentectomy)	Neoadjuvant BEP (4×)	CR (6 months)	2012
Lim et al. [31]	Abdominal distension	32 (female)	Asia (Korea)	11,577 (postoperatively)	No	Surgery (total abdominal hysterectomy with bilateral salpingo-oophorectomy, bilateral pelvic lymph nodes dissection, paraaortic lymph nodes sampling, total omentectomy, appendectomy)	Adjuvant BEP (6×)	CR (48 months)	2013
Lin et al. [32]	Abdominal discomfort	58 (female)	Asia (China)	2865 (preoperatively)	No	Omentectomy	Adjuvant BEP (4×)	CR (18 months)	2018
Fischerova et al. Current report	Abdominal bloating, nausea and vomiting, shortness of breath	37 (female)	Europe (Czech Republic)	496 (postoperatively)	Yes	Total abdominal hysterectomy with bilateral salpingo-ophorectomy was performed with pelvic peritonectomy, total omentectomy, partial diaphragmatic stripping and extirpation of abdominal peritoneum	Adjuvant BEP (4×)	CR (12 months)	2022

CR: complete response.

## Data Availability

Not applicable.

## Supplementary Materials

The following are available online at https://www.mdpi.com/article/10.3390/diagnostics12020304/s1, Figure S1: morphology assessment of tru-cut biopsy sample; Video S1: Peritoneal involvement; Figure S2: Immunoprofile of tru-cut biopsy sample; Figure S3: Tumor marker levels during chemotherapy.
