# Supplementary material for: Yolk Sac Tumor of the Omentum: A Case Report and Literature Review
Source: Diagnostics (Basel). 2022 Jan 25;12(2):304. doi: 10.3390/diagnostics12020304 (PMC8871053; doi:10.3390/diagnostics12020304)
Supplement: Supplementary file 1 [file diagnostics-12-00304-s001.zip › Supplementary figures.pdf]

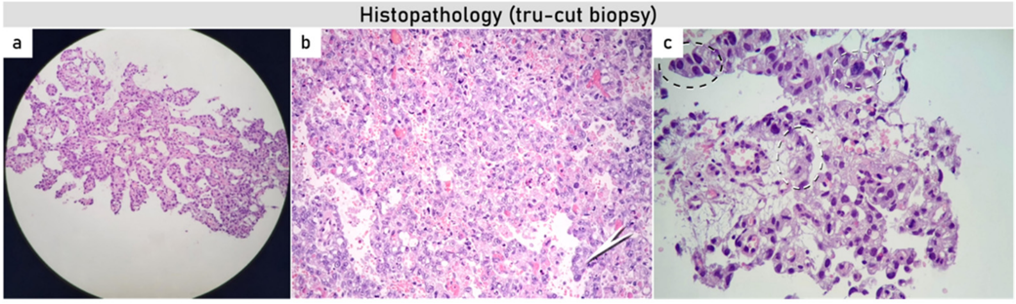

Figure S1. morphology assessment of tru-cut biopsy sample.

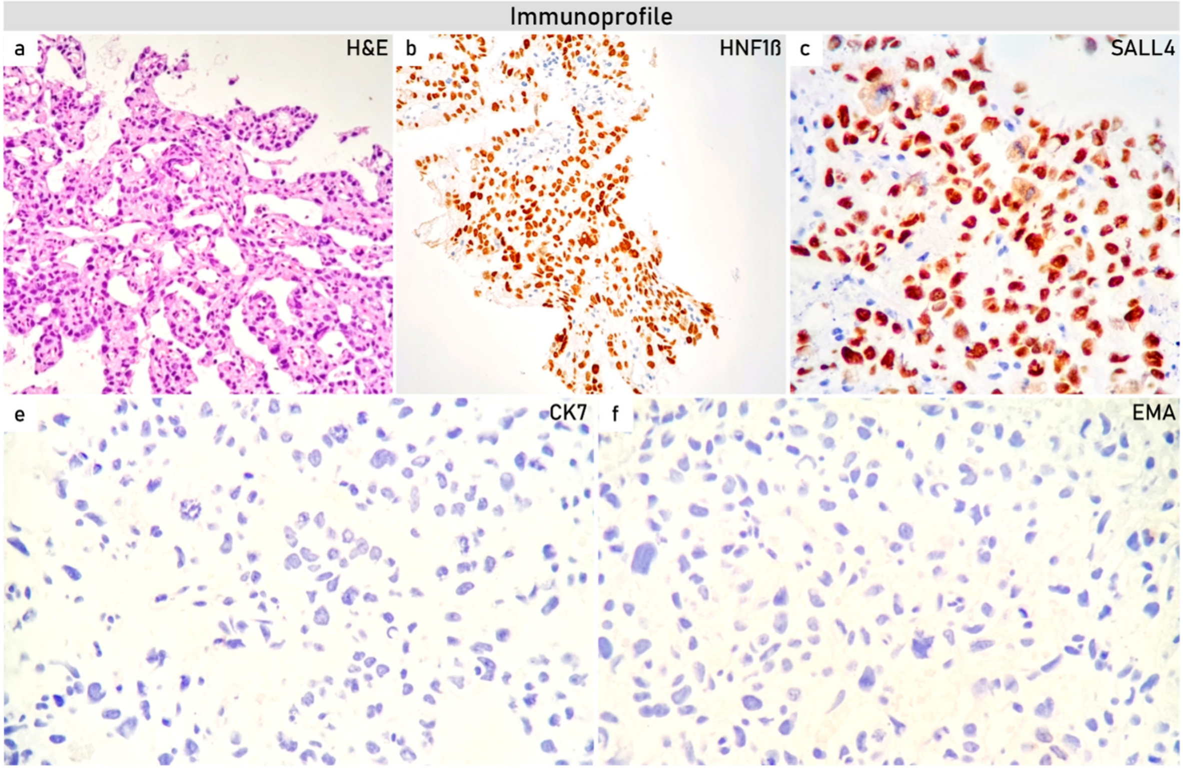

Figure S2. Immunoprofile of tru-cut biopsy sample.

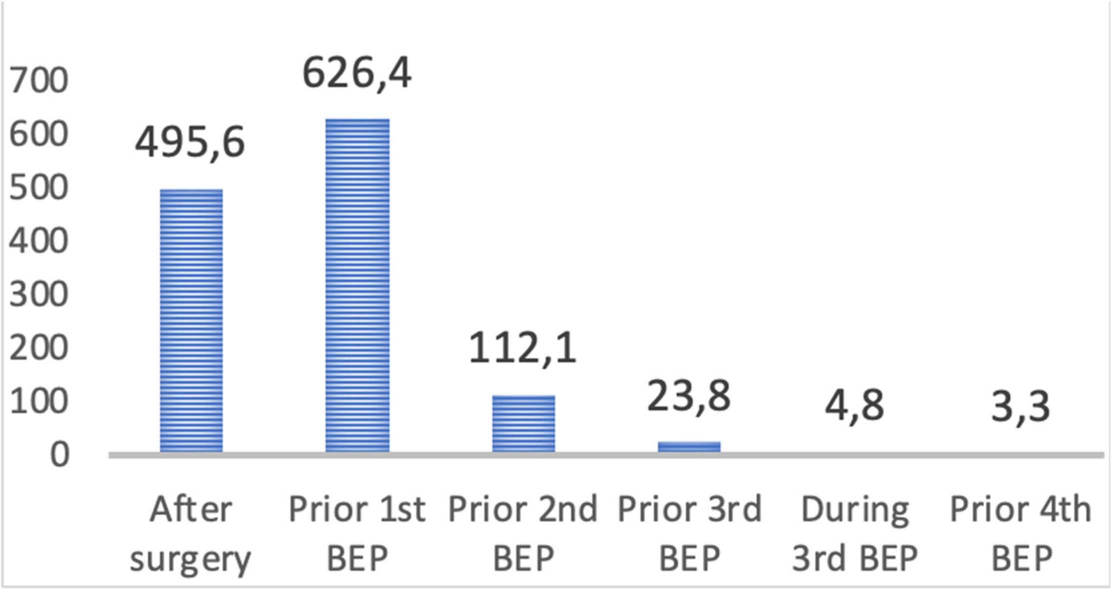

Figure S3. Tumor marker levels during chemotherapy.
